# Low Intensity, High Frequency Vibration Training to Improve Musculoskeletal Function in a Mouse Model of Duchenne Muscular Dystrophy

**DOI:** 10.1371/journal.pone.0104339

**Published:** 2014-08-14

**Authors:** Susan A. Novotny, Tara L. Mader, Angela G. Greising, Angela S. Lin, Robert E. Guldberg, Gordon L. Warren, Dawn A. Lowe

**Affiliations:** 1 Program in Physical Therapy and Rehabilitation Sciences, University of Minnesota, Minneapolis, Minnesota, United States of America; 2 Institute for Bioengineering and Bioscience, Georgia Institute of Technology, Atlanta, Georgia, United States of America; 3 Department of Physical Therapy, Georgia State University, Atlanta, Georgia, United States of America; Rutgers University -New Jersey Medical School, United States of America

## Abstract

The objective of the study was to determine if low intensity, high frequency vibration training impacted the musculoskeletal system in a mouse model of Duchenne muscular dystrophy, relative to healthy mice. Three-week old wildtype (n = 26) and *mdx* mice (n = 22) were randomized to non-vibrated or vibrated (45 Hz and 0.6 *g*, 15 min/d, 5 d/wk) groups. *In* *vivo* and *ex* *vivo* contractile function of the anterior crural and extensor digitorum longus muscles, respectively, were assessed following 8 wks of vibration. *Mdx* mice were injected 5 and 1 days prior to sacrifice with Calcein and Xylenol, respectively. Muscles were prepared for histological and triglyceride analyses and subcutaneous and visceral fat pads were excised and weighed. Tibial bones were dissected and analyzed by micro-computed tomography for trabecular morphometry at the metaphysis, and cortical geometry and density at the mid-diaphysis. Three-point bending tests were used to assess cortical bone mechanical properties and a subset of tibiae was processed for dynamic histomorphometry. Vibration training for 8 wks did not alter trabecular morphometry, dynamic histomorphometry, cortical geometry, or mechanical properties (P≥0.34). Vibration did not alter any measure of muscle contractile function (P≥0.12); however the preservation of muscle function and morphology in *mdx* mice indicates vibration is not deleterious to muscle lacking dystrophin. Vibrated mice had smaller subcutaneous fat pads (P = 0.03) and higher intramuscular triglyceride concentrations (P = 0.03). These data suggest that vibration training at 45 Hz and 0.6 *g* did not significantly impact the tibial bone and the surrounding musculature, but may influence fat distribution in mice.

## Introduction

Duchenne muscular dystrophy (DMD) is an X-chromosome-linked disease characterized by progressive muscle weakness [1], [2], [3]. Bone strength, or mechanical properties, are compromised in these patients as evident by the occurrence of fragility fractures upon falling from standing or sitting height [4], [5], [6], [7]. Compromised bone strength in DMD is multi-factorial, likely including effects of failure to accumulate peak bone strength during growth as well as declines in bone health secondary to the muscle disease. Furthermore, patients are recommended to avoid moderate- to high-intensity physical activity to prevent possible muscle damage and acceleration of the disease [8], [9], [10]. The absence of exercise, however, may result in the bone failing to increase in width, thus impacting bone strength. Preliminary data suggest that bone size is reduced in various skeletal sites in boys with DMD [11], [12], and those data are supported by reports that that these patients have low bone mass across their lifespan [4], [13]. Paralleling suboptimal attainment of bone strength, continual declines in muscle function associated with disease progression (i.e., reduced magnitude and frequency of muscle-induced mechanical loads) likely initiates disuse-mediated bone remodeling. This is supported by evidence that the discrepancies in bone mass between boys with DMD and their age-matched peers are accentuated with age, especially following the loss of ambulation where skeletal regions such as the hip and calcaneus experience dramatic bone loss [4], [13]. Therefore, effective bone-sparing interventions are warranted to thwart declines in bone health of boys with DMD in effort to preserve bone strength and prevent fractures.

Major determinants of bone health and interventions to preserve bone are related to mechanical loading [14]. Low-intensity loads (∼5–10 µε) applied thousands of times per day is hypothesized to be just as effective as high-intensity loads (≥1500 µε) applied a few times per day[15], [16]. Thus in the case of DMD, where high-intensity loads may be injurious to the inherently fragile muscle, utilizing low-intensity loads more often may be a reasonable approach to maintain bone health. Low intensity (i.e., ≤1.0 *g* of acceleration), high frequency vibration applies such stimulus to bone and has been shown to initiate an anabolic bone response [17], slow bone loss [18] [19], and improve bone mechanical properties [20]. Specifically, vibration has prevented bone loss associated with bed rest [21], as well as improved skeletal health in disabled children [22]. This suggests that vibration can have an osteogenic effect even in the presence of reduced mechanical loading (i.e., magnitude or spectrum of loads applied to the bone) or in the presence of disease. The benefits of vibration on skeletal muscle, however, remains ambiguous [21], [22], [23], [24], [25], [26], [27], [28], [29], and reports of contraindications raise concern [30], [31]. Consequently, vibration may be efficacious for bone health in patients with a muscle disease such as DMD; however it is important to confirm its simultaneous safety in skeletal muscle.

The *mdx* mouse is a widely used model of DMD, and like patients has alterations in bone health [32], [33], [34], [35], [36] and is relatively physically inactive over a 24-hr period particularly during active hours [37]. However, the mouse model is widely recognized to have a mild phenotype compared to boys with DMD, for instance *mdx* mice are non-distinguishable from wildtype mice in their ability to bear weight or locomote. *Mdx* mice, therefore, provide an appropriate model to determine the efficacy of low intensity, high frequency vibration to improve musculoskeletal function because while this function is compromised due to the disease, mice are fully capable of weight bearing during vibration bouts. The extent of bone’s response to vibration in mice is influenced by various factors including transmissibility of the vibration stimulus, the parameters of vibration used (i.e., acceleration and frequency), as well as genetic background of the mice [38], [39], [40]. These factors likely contributed to the lack of vibration-induced alterations in trabecular [41], [42], [43] and cortical bone [14], [44], [45]; highlighting that parameters of vibration are not universally effective across all mice. Therefore, ‘optimization’ specific to the model of interest may be necessary to maximize musculoskeletal benefits. Recently, we compared six different pairs of vibration parameters and identified 45 Hz at 0.6 *g* to best initiate increased expression of osteogenic genes in male *mdx* mice aged 5–7 weeks at the mRNA level [46]. It remains to be determined if those acute increases in gene expression would translate to improved bone structure and function with prolonged vibration training in dystrophic mice.

The objective of the present study, therefore, was to determine the extent to which low intensity, high frequency vibration training impacted the musculoskeletal system in mice modeling DMD, relative to healthy mice. Specifically, we sought to determine if trabecular morphometry, cortical geometry, and mechanical properties are better in tibia of vibrated than non-vibrated mice. Previous studies in mice showed that at least 3–6 weeks of vibration training is necessary to evoke structural adaptations within bone [39], [44], [47], [48], [49]. Consequently, we hypothesized that 8 weeks of vibration would improve the tibial bone of *mdx* mice. Specifically, three-point bending tests were utilized at the mid-diaphysis of the tibia to assess changes in mechanical properties, and micro-computed tomography (µCT) was performed to elucidate the possible underlying mechanical determinants of altered strength (i.e., geometry, mechanical properties and intrinsic material properties). Dynamic histomorphometry was also used as a direct measure of osteoblast activity in tibiae from *mdx* mice. In addition, we hypothesized that vibration training would not be injurious to dystrophic muscle as indicated by assessments of anterior crural muscle strength, contractility of extensor digitorum longus (EDL) muscle, muscle morphology, and plasma creatine kinase activity.

## Methods

### Animals and Experimental Design

Male wildtype (C57Bl/10) and *mdx* mice were obtained from our SPF-maintained breeding colony at the University of Minnesota. Mice were housed in standard cages, 3–4 mice per cage, on a 12∶12-h light-dark cycle at 20–23°C and were provided food and water ad libitum. Mice were randomly assigned to either a non-vibrated group (wildtype non-vibrated n = 12, *mdx* non-vibrated n = 11) or vibration group (wildtype vibrated n = 14, *mdx* vibrated n = 11). Mice allocated to the vibration groups were exposed to 15-min bouts of vibration 5 d/wk for 8 wk (range 55–58 d) starting when mice were 3 wk of age. The vibration stimulus consisted of a 45-Hz stimulus with 0.6 *g* of acceleration (where 1 *g* is equivalent to the acceleration due to gravity) based on our preliminary work in *mdx* mice [46]. This vibration stimulus was well tolerated by *mdx* mice as previously reported [46] as well as for wildtype mice [50]. Specifically, in this study behaviors, ambulation patterns, and activities were indistinguishable between genotypes during (see Video S1 and S2) and immediately after bouts of vibration. The height of the vibration cage was set to 5 cm, to limit rearing and ensure mice consistently bore weight on their hindlimbs during the entire bout of vibration. This was verified during each vibration bout as mice were continually monitored by an investigator. The combination of these factors gives us confidence that an equivalent vibration stimulus was transmitted to the bone of *mdx* and wildtype mice.

Relatively young mice were selected for this study in order to determine the impact of prolonged vibration training while the disease pathology in this mouse model is apparent (i.e., 3–12 wk of age in *mdx* mice). *Mdx* mice, unlike boys with DMD, do not have progressive muscle pathology past the age of about 12 weeks, thus limiting the ages in which the mouse model mimics the disease. Mice in the non-vibrated group were placed on the vibration platform for the same duration of time, but with the machine turned off.

*Mdx* mice were injected subcutaneously with 15 mg/kg body mass (BM) Calcein (Sigma, St. Louis, MO) 5 days prior to sacrifice, and 1 day prior to sacrifice with 90 mg/kg BM Xylenol orange (Sigma) to quantify dynamic trabecular bone histomorphometry, as adapted from [51]. At 11 wk of age, mice were sacrificed by first anesthetizing with a cocktail of: fentanyl citrate (0.2 mg/kg body mass (BM)), droperidol (10 mg/kg BM) and diazepam (5 mg/kg BM). Plasma was collected via retro-orbital bleed and flash frozen in liquid nitrogen to assess creatine kinase activity. Functional capacity of the left anterior crural muscles (i.e., tibialis anterior (TA), extensor digitorum longus (EDL), and extensor hallucis longus muscles) were then assessed *in* *vivo* by quantifying maximal isometric torque and susceptibility to contraction-induced injury. The anterior crurals were selected because we previously showed vibration training to improve contractility of this muscle group [50]. Immediately following *in* *vivo* analyses, mice were injected with supplemental anesthesia intraperitoneal (i.e., 75 mg/kg BM sodium pentobarbital for wildtype mice and 37.5 mg/kg BM for *mdx* mice). The EDL muscle from the right hindlimb was excised and used to assess *ex* *vivo* force-producing capacity. This muscle was chosen because in *mdx* mice it is sensitive to disease progression, eccentric contraction-induced injury, and can adapt in response to intervention [52], [53]. Prior to exsanguination, TA, soleus, and gastrocnemius muscles were also excised and weighed. These muscles were selected due to their proximity to the vibration platform and hence their potential ability to be affected by vibration training.

The subcutaneous and visceral fat pads were also excised and weighed, as consistent reductions in fat pad masses have been reported following long-term vibration training [47], [50], [54]. The TA, EDL, gastrocnemius, and soleus muscles were dissected and snap frozen in liquid nitrogen or mounted in Tissue-Tek OCT (Sakura, Torrance, CA). Tibial bones were removed and stored in either phosphate-buffered saline at –20°C until the time of mechanical testing or in 70% alcohol at 4°C until the time of dynamic histomophometric processing. The tibial bone was selected, rather than the femur, due to its proximity to the vibration plate. That is, the range of transmissibility of vibration stimulus is reduced with increasing distance from the platform [55], [56], and consequently, bone’s response to vibration may be more robust in the tibia compared to the femur.

This study was carried out in strict accordance with the recommendations in the Guide for the Care and Use of Laboratory Animals of the National Institutes of Health and all procedures were approved by the Institutional Animal Care and Use Committee at the University of Minnesota (Permit Number: 1109A04549). Anesthetic regimes used were recommended and approved by veterinarian staff. For each of the musculoskeletal assessments, one investigator performed that specific assessment on all mice and all investigators were blinded to the genotype and training group of each mouse when performing the assessments.

### In Vivo Assessments of Anterior Crural Muscle Functional Capacity

Mice underwent *in* *vivo* contractile testing of the anterior crural muscles of the left hindlimb. Outcome measures of interest included peak isometric dorsiflexor torque production [57] and peak eccentric and isometric torque loss following contraction-induced injury [53], [58]. Muscle injury was induced as previously described [59], by performing 100 electrically-stimulated eccentric contractions evoked using 250 Hz at a constant optimal voltage, with an angular excursion of 38 degrees at an angular velocity of 2000 degrees per second with the exception of 12 seconds between contractions. Five minutes following the last eccentric contraction, peak isometric torque was re-assessed.

### Ex Vivo Assessments of EDL Muscle Contractility

Contractile measurements of isolated EDL muscles included peak twitch force, time-to-peak twitch force, twitch one-half relaxation time, peak isometric tetanic force (P_o_), maximal rates of tetanic force production and relaxation, peak eccentric force, and percent decline in isometric tetanic force following eccentric contraction-induced injury [60]. Eccentric contraction-induced injury consisted of five eccentric contractions with 3 minutes in between contractions. Eccentric contractions were evoked by passively shortening the EDL muscle from its anatomical muscle length (L_0_) to 0.95L_o_, and then simultaneously stimulating the muscle for 133 ms as the EDL muscle lengthened to 1.05L_o_ at a rate of 0.75L_o_/s [53]. EDL muscles were trimmed, blotted dry, and weighed immediately following the measurements. Physiological cross-sectional area was calculated using EDL muscle mass, L_o_, and a fiber length-to-muscle length ratio of 0.44 [60], [61]. Specific P_o_ was determined by dividing P_o_ by the calculated physiological cross-sectional area of the muscle.

### Muscle Morphology

Altered vascularity within the soleus muscle has been noted following vibration training [30], [50], therefore we measured capillary density at the distal end and mid-belly of the soleus muscles. Capillary density was quantified by counting the number of capillaries surrounding a fiber for 200 fibers per muscle following staining by a periodic acid Schiff reaction [50]. Central nucleated fibers (i.e., a marker of muscle damage and regeneration) were also assessed at the distal end and mid-belly of the soleus muscle as well as the mid-belly of the TA muscle. The number of central nucleated fibers present per 300 fibers was counted in each of these regions from hematoxylin and eosin-stained sections [37].

### Intramuscular Triglyceride Concentration

Smaller fat pads are consistently reported following long-term vibration training [47], [50], [54], therefore to extend these results further, we wanted to see what effect vibration has on intramuscular fat. We chose to measure triglyceride concentration within the gastrocnemius muscle, as this method has been previously utilized to assess triglycerides in liver, serum, and fat pads following vibration training [47]. Intramuscular triglycerides were extracted and isolated from gastrocnemius muscles as previously described [62]. Briefly the muscles were homogenized in 20 volumes of a 2∶1 chloroform-methanol mixture. The homogenate was vortexed and washed with a volume of saline necessary to obtain an 8∶4∶3 ratio of chloroform, methanol, and water. The homogenate was centrifuged at 1160 *g* for 20 minutes to obtain a biphasic separation. A 500-µl sample of the lower phase was removed, transferred to a new tube, dried under nitrogen gas, and resuspended in 250 µL of phosphate-buffered saline containing 1% Triton X-100. Triglyceride concentrations were determined using an enzymatic colorimetric assay employing glycerol-3-phosphate oxidase (Cat. #461-08992; Wako Pure Chemical Industries, Ltd. Richmond, VA). Triglyceride concentration is expressed as milligrams per gram of wet muscle mass.

### µCT of Tibial Bone Metaphysis and Mid-diaphysis

A µCT system (Scanco Medical microCT 40, Bruttisellen, Switzerland) was used to quantify trabecular morphometry in the tibial metaphysis as well as cortical bone geometry and volumetric bone density (vBMD) at the tibial mid-diaphysis [34]. Trabecular bone morphometry was assessed in the proximal tibial metaphysis (50 slice region of interest, starting 60 µm distal to the last image containing the growth plate, using 12-µm voxel size) as previously described [34]. Bone volume fraction (BV/TV), trabecular thickness, trabecular number, trabecular separation and trabecular vBMD were determined for each slice and the average value across each of the 50 slices was used for statistical analyses.

The following outcome measures were assessed in the transverse plane on the central 0.8-mm region of the tibial diaphysis: cortical cross-sectional area, cortical thickness, periosteal diameter, cross-sectional moment of inertia (CSMI), and vBMD. CSMI about the anterior-posterior axis corresponds to the CSMI about the bone-bending axis during three-point bending tests. These measures were assessed for each of the 66 slices within the 0.8 mm region of the tibial diaphysis, and the average for all 66 slices was used for statistical analyses. Following the completion of imaging, tibial bones were refrozen in PBS until the time they underwent mechanical testing.

### Mechanical Testing of the Tibial Mid-diaphysis

Mechanical testing procedures for assessing the functional capacity of the mouse tibial bone has previously been described in detail [34], [63], [64], [65]. Briefly, the left tibial bone of each mouse was placed on its lateral side in a Mecmesin MultiTest 1-D test machine, and was loaded in three-point bending at the mid-diaphysis using a Mecmesin AFG-25 load cell (Mecmesin, West Sussex, UK). The functional capacity of the tibial bone was quantified by ultimate load, stiffness, and deflection and energy absorbed to ultimate load using custom designed TestPoint software (TestPoint version 7; Measurement Computing Corp.) [34], [65].

### Trabecular Bone Dynamic Histomorphometry

A subset of the tibiae were dehydrated and embedded without demineralization in methyl-methacrylate (Fisher Scientific, Pittsburgh, PA) as previously described [66]. Briefly, 5-µm thick longitudinal sections were cut on a microtome (Leica, Heidelberg, Germany) and mounted unstained. Fluorochrome labels were visualized at 20x, and dynamic histomorphometric measures were made using OsteoMeasure image analyzer (OsteoMetric, Atlanta, GA) in a region 60 µm distal to the proximal growth plate. Outcome measures of interest include mineralized surface per bone surface, mineral apposition rate, and bone formation rates relative to bone surface or total volume.

### Statistical Analyses

Power calculations determined that 10 mice per group were necessary to detect significant group differences with two-way ANOVAs with a minimum power of 80% and α-level of 0.05. The effects of vibration (45 Hz at 0.6 *g* vs. non-vibrated) and genotype (wildtype vs. *mdx*) were assessed by two-way ANOVAs with vibration and genotype as the fixed factors. Eccentric contraction data were assessed by three-way repeated measure ANOVAs with vibration, genotype and contraction numbers as the fixed factors. When significant interactions were present, Holm-Sidak post-hoc measures were used to determine differences among the groups. When assumptions of normality or equal variance were violated, Kruskal-Wallis One Way Analysis of Variance on Ranks was performed along with Dunn’s post-hoc measures. Dynamic histomorphometry measures of the tibia were only performed on *mdx* non-vibrated and vibrated mice, and therefore the data were assessed by t-tests. All statistical analyses were carried out using SigmaPlot version 11.0 (Systat Software Inc; Point Richmond, CA).

## Results

### Body, Muscle and Fat Pad Masses and Intramuscular Triglyceride Content

**Figure 1:**
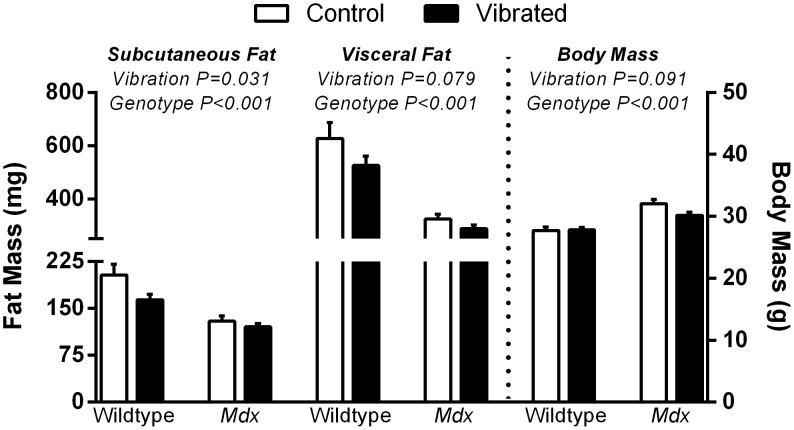
Eight weeks of vibration training affected fat pad masses but not body masses. Vibrated mice had smaller sized subcutaneous fat pads following 8-weeks of training. *Mdx* mice had a larger body mass but smaller fat pad masses compared to wildtype mice following 8-weeks of training. Body masses were not different in mice subject to vibration compared to non-vibrated control mice. Data are means ± SE. P-values associated with the main effects of two-way ANOVAs are indicated above each set of bars. Interactions between vibration and genotype P≥0.056.

At the start of the study, body mass did not differ between vibrated and non-vibrated groups (P = 0.654), though *mdx* mice weighed less than wildtype at 3-wks of age (9.0±0.3 vs 10.7±0.7 *g*, P = 0.005). Eight weeks later vibrated mice tended to have lower body mass than non-vibrated mice and *mdx* mice were 12% heavier (Figure 1). *Mdx* mice also had greater muscle masses than wildtype mice (Table 1). Tibialis anterior, soleus, and gastrocnemius muscle masses were not impacted by vibration (Table 1). Vibrated mice had 5% smaller EDL muscles, primarily due to vibrated *mdx* mice having 9% smaller EDL muscles compared to non-vibrated *mdx* mice (Table 1).

**Table 1:** Effects of low intensity, high frequency vibration training and genotype on muscle and muscle fiber characteristics.

	WildtypeNon-vibrated	WildtypeVibrated	*Mdx*Non-vibrated	*Mdx* *Vibrated*	P-values for Two-Way ANOVA		
					Main effectof Vibration	Main effect ofGenotype	Interaction(Vibration×Genotype)
**Extensor Digitorum Longus Muscle**							
Mass (mg)	11.6 (0.3)	11.6 (0.2)	16.2 (0.6)	14.7 (0.4)	0.045	<0.001	0.054
Anatomical Muscle Length (mm)	12.6 (0.1)	12.5 (0.1)	12.7 (0.1)	12.6 (0.1)	0.516	0.411	0.811
**Tibialis Anterior Muscle**							
Mass (mg)	45.3 (1.2)	48.0 (1.3)	78.1 (2.9)	75.4 (3.3)	0.991	<0.001	0.238
Centrally Nucleated Fibers, Mid-belly (%)	1.6 (0.6)	3.3 (0.9)	71.2 (1.5)	70.3 (1.2)	0.741	<0.001	0.298
**Soleus Muscle**							
Mass (mg)	6.7 (0.4)	7.0 (0.3)	9.3 (0.6)	8.3 (0.4)	0.405	<0.001	0.183
Centrally Nucleated Fibers, Mid-belly (%)	2.5 (0.4)	5.0 (1.7)	63.6 (2.2)	64.2 (2.7)	0.457	<0.001	0.636
Centrally Nucleated Fibers, Distal (%)	8.7 (7.2)	8.3 (5.5)	62.4 (6.7)	62.7 (3.2)	0.991	<0.001	0.950
Capillaries per Fiber, Mid-belly	2.6 (0.1)	2.9 (0.2)	2.5 (0.2)	2.7 (0.2)	0.174	0.258	0.832
Capillaries per Fiber, Distal	2.5 (0.1)	2.8 (0.2)	2.4 (0.1)	2.5 (0.1)	0.315	0.208	0.598
**Gastrocnemius Muscle**							
Mass (mg)	125.9 (5.2)	128.6 (4.2)	173.0 (9.0)	164.8 (4.3)	0.654	<0.001	0.363
Triglyceride Concentration (mg/g)	0.889 (0.066)	1.179 (0.116)	0.906 (0.098)	1.085 (0.108)	0.025	0.696	0.577

Values are means (SE).

Vibrated mice also had significantly smaller subcutaneous fat pads and tended to have lower visceral fat pad masses compared to non-vibrated mice (Figure 1). Main effects of genotype were consistently present for fat pad masses with *mdx* mice having up to 47% less fat pad masses (Figure 1). Vibrated mice had 26% higher concentrations of triglycerides per gram of wet gastrocnemius muscle mass (Table 1). Triglyceride concentrations were not different between genotypes (Table 1).

### Muscle Morphology

Vibration had no impact on capillary density and percentage of centrally-nucleated muscle fibers in either the mid-belly or distal end of the soleus muscle (Table 1). *Mdx* mice had more centrally-nucleated fibers in soleus and tibialis anterior muscles compared to those of wildtype mice (Table 1).

### In Vivo Assessments of Anterior Crural Muscle Functional Capacity

**Figure 2:**
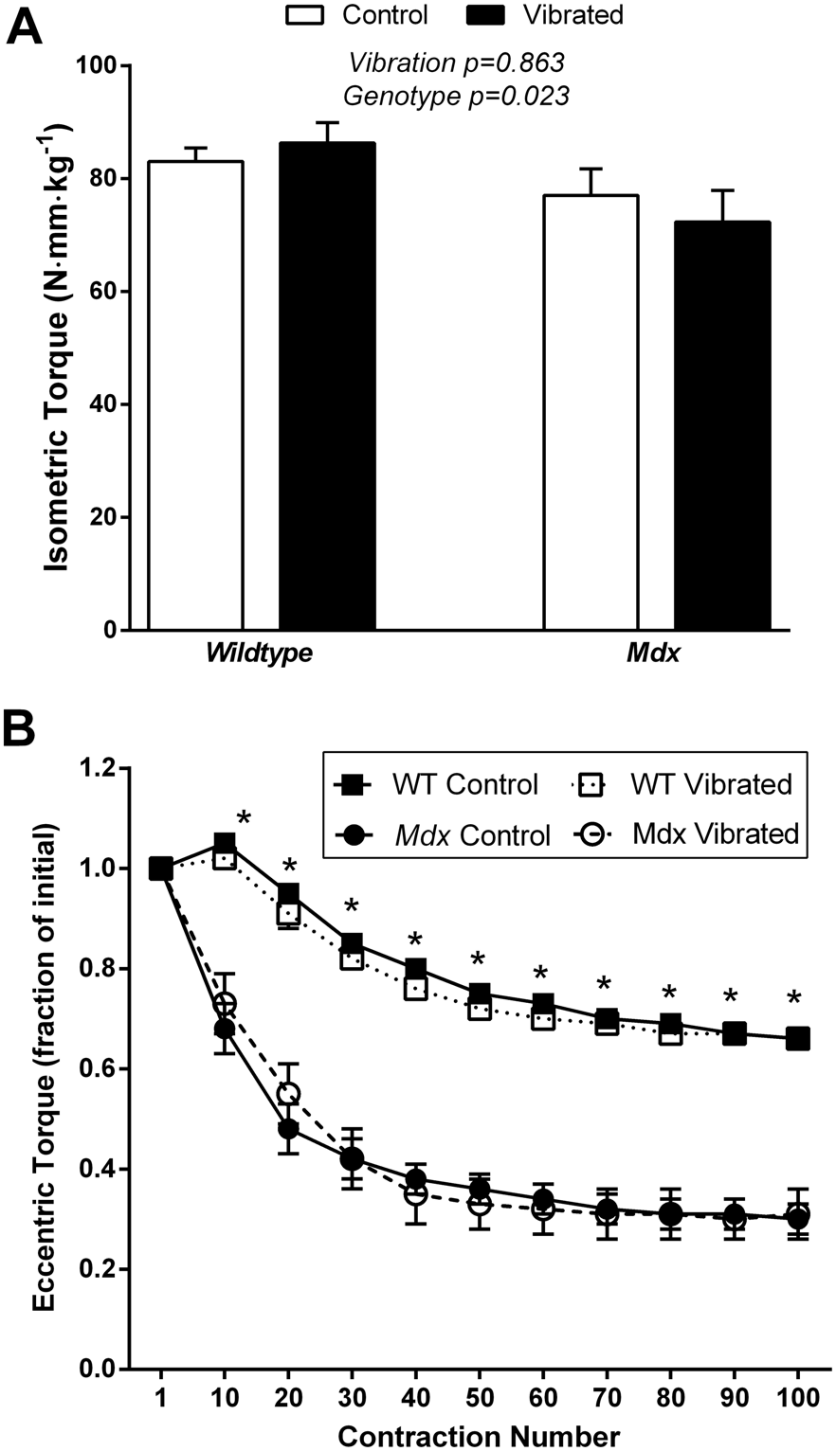
Eight weeks of vibration did not impact *in* *vivo* muscle strength or susceptibility to injury. **A)** Maximal isometric torque was not different between vibrated and non-vibrated mice following 8-weeks of training; isometric torque was less in *mdx* than wildtype mice. Interaction between vibration and genotype P≥0.357. **B)** Vibration training for 8 weeks did not alter susceptibility to eccentric contraction-induced injury. As expected, *mdx* mice were more susceptible to eccentric injury relative to wildtype mice. Data are means ± SE. In Panel A, P-values associated with the main effect of two-way ANOVAs are indicated above the bars. In panel B, only a main effect of genotype was present, where * signifies a significant (P<0.05) difference between *mdx* and wildtype mice at that contraction number.

To determine if vibration training affected skeletal muscle tissue in close proximity to the vibrating platform, dorsiflexor torque was assessed. Overall, the contractility measures of anterior crural muscles showed no effect of vibration. Peak isometric dorsiflexor torque and peak isometric torque normalized to body mass were not impacted by vibration (Table 2 and Figure 2A, respectively), indicating that muscle strength was not altered following vibration training. Genotype differences in isometric torque production were only apparent after accounting for the greater body mass of the *mdx* mice (Figure 2A). Susceptibility to contraction-induced injury, as indicated by the decline in peak eccentric torque over a series of 100 eccentric contractions (Figure 2B) and isometric torque loss (Table 2), was not affected by vibration. *Mdx* mice had a substantial loss of anterior crural muscle functional capacity following eccentric injury as indicated by a ∼70% decline in peak eccentric torque vs. only 34% decline for wildtype mice (Figure 2B), and a larger isometric torque loss (Table 2). These data indicate that lack of dystrophin, but not vibration, is detrimental to muscle function. Similarly, plasma creatine kinase activity did not differ between vibrated and non-vibrated groups (P = 0.974), but was 4-fold higher in *mdx* than wildtype mice (4507+/−200 U/L vs. 1055+/−210 U/L, P<0.001).

**Table 2:** Muscle contractile measures following 8 weeks of low intensity, high frequency vibration training in wildtype and *mdx* mice.

	WildtypeNon-vibrated	WildtypeVibrated	*Mdx*Non-vibrated	*Mdx* *Vibrated*	P-values for Two-Way ANOVA			
					Main effect ofVibration	Main effect ofGenotype		Interaction(Vibration×Genotype)
***In Vivo*** ** Function of Anterior Crural Muscles**								
Maximal Isometric Torque (N•mm)	2.31 (0.10)	2.42 (0.10)	2.45 (0.16)	2.18 (0.17)	0.526	0.714	0.164	
Isometric Torque Loss Following EccentricContractions (%)	42.3 (1.9)	41.5 (1.7)	71.9 (5.1)	61.6 (6.9)	0.192	<0.001	0.257	
**Ex Vivo Function of EDL Muscles**								
Peak Twitch Force (mN)	99.3 (3.5)	94.0 (2.2)	94.4 (3.1)	90.2 (3.1)	0.118	0.150	0.860	
Time-to-Peak Twitch Force (ms)	19.0 (0.3)	18.8 (0.3)	19.1 (0.5)	20.1 (0.5)	0.276	0.071	0.108	
Half-Relaxation Time of Twitch Force (ms)	15.0 (0.4)	13.9 (0.4)	17.8 (0.3)	18.3 (0.7)	0.558	<0.001	0.133	
Maximal Rate of Tetanic Force Development (N•s^−1^)	10.9 (0.4)	10.9 (0.3)	10.2 (0.4)	10.4 (0.5)	0.784	0.167	0.738	
Maximal Rate of Tetanic Force Relaxation (N•s^−1^)	22.6 (0.4)	23.4 (0.7)	18.5 (1.0)	16.5 (1.2)	0.477	<0.001	0.116	
Peak Eccentric Force (mN)	639.0 (13.3)	629.6 (15.1)	549.2 (19.2)	517.6 (22.7)	0.253	<0.001	0.533	
Isometric Force Loss Following EccentricContractions (%)	4.1 (1.0)	3.8 (1.4)	63.1 (5.5)	67.5 (5.7)	0.617	<0.001	0.553	

Values are means (SE). Isometric torque loss was calculated as the percent difference between isometric torque measured before and after the 100 eccentric contractions. Isometric force loss was calculated as the percent difference between peak isometric tetanic force measured before and after the 5 eccentric contractions.

### Ex Vivo Assessments of EDL Muscle Contractility

**Figure 3:**
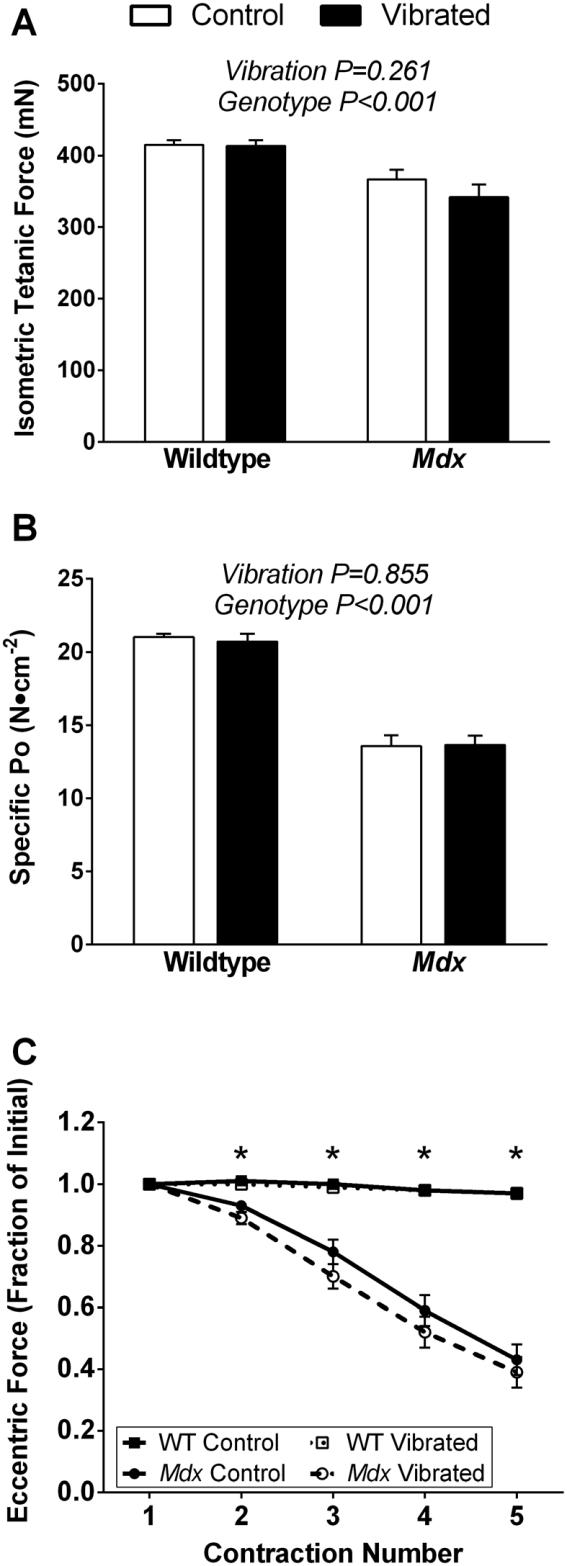
Eight weeks of vibration training did not impact *ex* *vivo* EDL muscle contractile function. Vibration training for 8 weeks did not influence the following EDL muscle contractile measures: **A)** maximal isometric tetanic force production, **B)** specific force, or **C)** susceptibility to eccentric contraction-induced injury compared to non-vibrated mice. As expected, *mdx* mice had lower values for each of the three measurements of EDL muscle function compared to wildtype mice. Data are means ± SE. P-values associated with the main effects of two-way ANOVAs are indicated above each set of bars in Panel A and B. In panel C, an interaction between genotype and eccentric contraction number was present, where the * signifies a significant (P<0.05) difference between *mdx* and wildtype mice from post-hoc testing. Interactions between vibration and genotype for panels A and B P≥0.329.

Force-generating capacity of the EDL muscle assessed *ex* *vivo* was not affected by 8 weeks of vibration training. Vibration had no impact on peak twitch force, maximal isometric tetanic force, specific P_o_, peak eccentric force, and eccentric or isometric force loss following contraction-induced injury (Figure 3 and Table 2). Characteristics relating to speed of EDL muscle contraction, including time-to-peak twitch force, half-relaxation time of twitch force, and maximal rates of tetanic force development and relaxation were also not effected by vibration (Table 2). Most of the EDL contractile measures were different between wildtype and *mdx* mice, reflecting the expected pathology of the muscle disease (Figure 3 and Table 2).

### µCT of Tibial Bone Metaphysis and Mid-diaphysis

**Figure 4:**
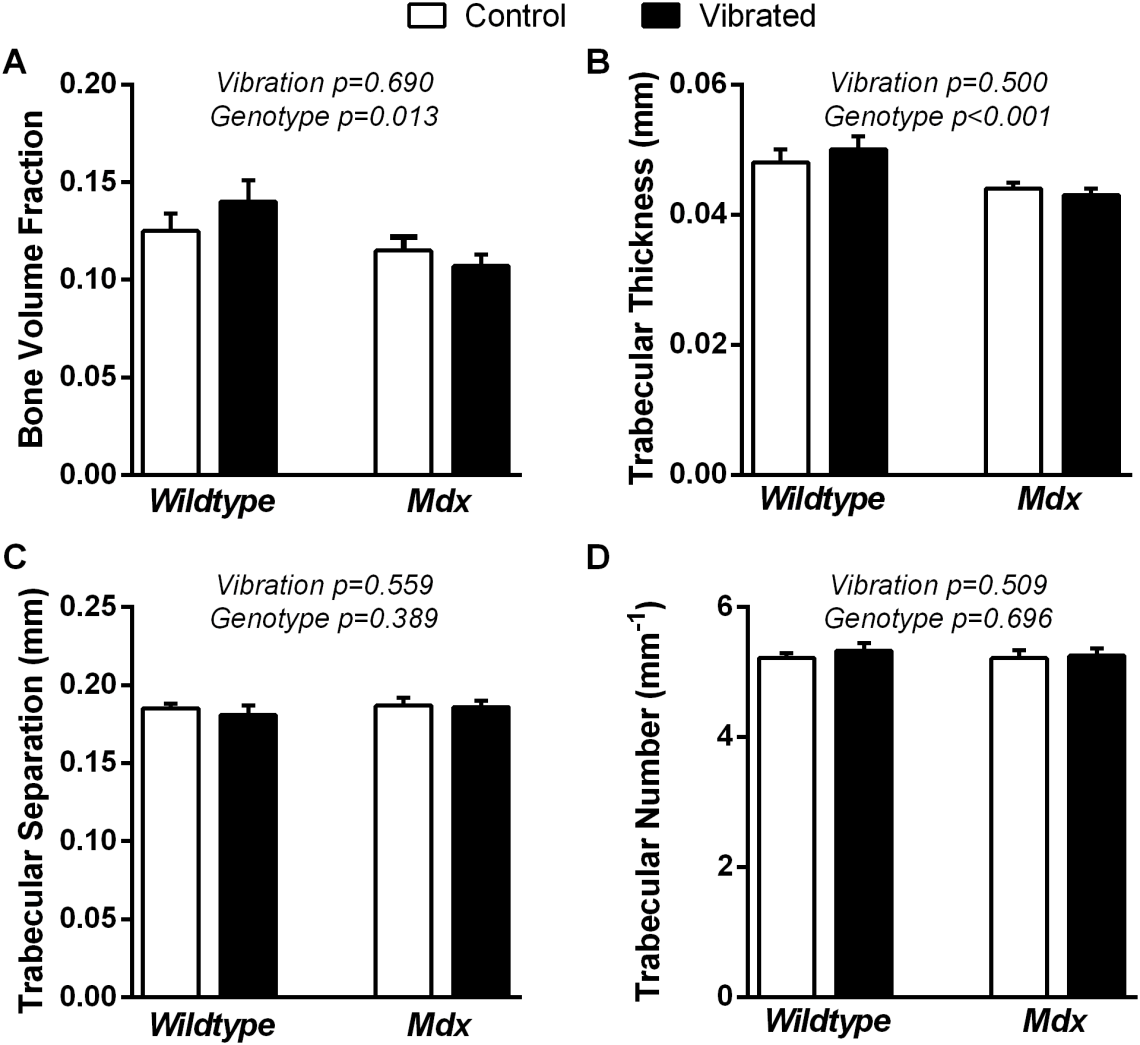
Eight weeks of vibration training did not impact trabecular bone in the tibia. Vibration training for 8 weeks did not influence trabecular bone **A)** volume fraction, **B)** thickness, **C)** separation, or **D)** number. As expected, *mdx* mice had lower values for trabecular bone volume fraction and thickness compared to wildtype mice. Data are means ± SE. P-values associated with the main effects of two-way ANOVAs are indicated above each set of bars. Interactions between vibration and genotype was P≥0.165.

µCT was performed to determine the extent to which vibration and genotype influenced trabecular bone morphometry and cortical bone geometry at the proximal metaphysis and mid-diaphysis, respectively. In the proximal metaphysis of the tibia, vibration did not influence trabecular morphometry, though differences between *mdx* and wildtype were detected (Figure 4). Specifically, bone volume fraction and trabecular thickness, number, and separation did not differ between non-vibrated and vibrated mice (Figure 4). The lack of altered trabecular morphometry in the metaphysis of *mdx* mice, following vibration, was confirmed by dynamic histomorphometry. Overall, vibration had no impact on bone formation in *mdx* mice as indicated by the average mineralized surface per bone surface (34.1±1.8% for vibrated mice and 34.1±2.1% for non-vibrated mice, P = 0.989), mineral apposition rate (1.04±0.04 µm/d for vibrated mice and 1.08±0.03 µm/d for non-vibrated mice, P = 0.373), bone formation rate per bone surface (0.36±0.03 µm^3^/µm^2^/d for vibrated mice and 0.37±0.03 µm^3^/µm^2^/d for non-vibrated mice, P = 0.633) or bone formation rate per tissue volume (0.192±0.016%/d for vibrated mice and 0.194±0.018%/d for non-vibrated mice, P = 0.908). For the differences in trabecular bone morphometry across genotypes, bone volume fraction showed that *mdx* mice had less bone than wildtype (0.111±0.006 for *mdx* mice and 0.133±0.006 for wildtype mice), which was attributed to having 12% thinner trabeculae (Figure 4B). Trabecular separation and number were not influenced by genotype (Figure 4C and D, respectively).

**Figure 5:**
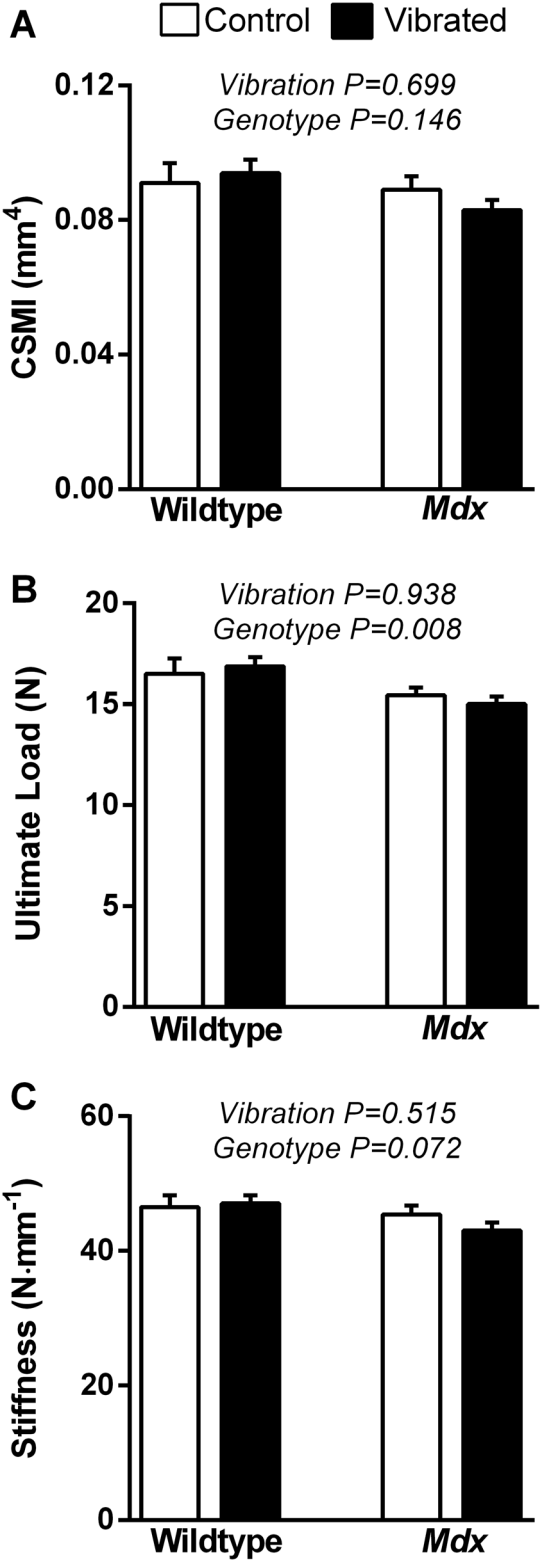
Eight weeks of vibration training did not impact tibial cortical bone. Vibration training for 8 weeks did not influence the following tibial cortical bone properties: **A)** cross-sectional moment of inertia, **B)** ultimate load, or **C)** stiffness. *Mdx* mice had lower values for ultimate load and trends for lower stiffness compared to wildtype mice. Data are means ± SE. P-values associated with the main effects of two-way ANOVAs are indicated above each set of bars. Interactions between vibration and genotype P≥0.287.

Neither vibration nor genotype influenced any parameter of cortical bone geometry at the tibial mid-shaft (Table 3 and Figure 5A). These data suggest that the shape of the bone was similar across all groups, despite the tendency of *mdx* mice to have longer tibial lengths (Table 3).

**Table 3:** Effects of low intensity, high frequency vibration training on tibial bone cortical geometry, mechanical properties, and intrinsic material properties in wildtype and *mdx* mice.

	WildtypeNon-vibrated	WildtypeVibrated	*Mdx*Non-vibrated	*Mdx* *Vibrated*	P-values for Two-Way ANOVA		
					Main effect ofVibration	Main effect ofGenotype	Interaction(Vibration×Genotype)
Tibial Length (mm)	17.73 (0.08)	17.89 (0.07)	17.96 (0.06)	17.92 (0.05)	0.409	0.059	0.154
**Cortical Geometric Properties**							
Cortical Cross-Sectional Area (mm^2^)	0.76 (0.03)	0.79 (0.02)	0.77 (0.02)	0.75 (0.02)	0.818	0.610	0.274
Cortical Thickness (mm)	0.22 (0.01)	0.23 (0.00)	0.23 (0.00)	0.23 (0.00)	0.336	0.495	0.429
**Mechanical Functional Properties**							
Energy to Ultimate Load (mJ)	4.05 (0.28)	4.26 (0.10)	3.53 (0.11)	3.59 (0.27)	0.521	0.005	0.720
Deflection to Ultimate Load (mm)	0.45 (0.01)	0.48 (0.02)	0.43 (0.01)	0.45 (0.03)	0.148	0.142	0.921
**Intrinsic Material Properties**							
Ultimate Stress (MPa)	282.8 (5.3)	279.9 (3.5)	273.6 (6.4)	280.0 (5.2)	0.746	0.397	0.385
Modulus of Elasticity (GPa)	10.7 (0.3)	10.6 (0.3)	10.8 (0.4)	10.9 (0.3)	0.945	0.620	0.713
Cortical vBMD (mg•cm^−3^)	1345.1 (17.7)	1351.8 (9.0)	1303.7 (13.9)	1313.2 (10.8)	0.543	0.004	0.918
Trabecular vBMD (mg•cm^−3^)	1095.6 (3.1)	1094.1 (4.1)	1077.8 (3.3)	1079.4 (4.3)	0.986	<0.001	0.679

Values are means (SE) vBMD, volumetric bone mineral density.

### Mechanical Testing of the Tibial Mid-diaphysis

Three-point bending tests were performed at the mid-shaft of the tibial diaphysis to determine if cortical bone mechanical properties were affected, even in the absence of change in cortical bone geometry. The ultimate load and stiffness of tibial bones were not different between vibrated and non-vibrated mice (Figure 5B and C). Energy and deflection to ultimate load were also not different between vibrated and non-vibrated mice (Table 3). Comparisons across genotypes confirmed that mechanical properties of the tibial bone were compromised in *mdx* mice, as indicated by 9% smaller ultimate loads and a trend toward lower tibial stiffness (Figure 5B and C), as well as a significantly lower energy absorption to ultimate load compared to wildtype mice (Table 3).

Overall, vibration had no impact on any measure of intrinsic material properties of the tibia (Table 3). While ultimate stress and modulus of elasticity values were similar across genotypes, µCT revealed differences in vBMD between *mdx* and wildtype mice at both the tibial proximal metaphysis (trabecular) and the tibial mid-diaphysis (cortical) with *mdx* mice having up to 3% lower vBMD (Table 3).

## Discussion

Vibration training has been reported to enhance bone and muscle in humans and rodent models in some, but not all studies. Our study failed to show any enhancement in either of these two tissues. First, 8 weeks of low intensity vibration training did not alter trabecular morphology, cortical bone geometry, or cortical bone mechanical properties in tibia of wildtype mice or mice modeling Duchenne muscular dystrophy. Secondly, vibration did not alter any of our measures of contractile function or histology in lower hindlimb muscles. Despite the lack of benefit, it is noteworthy that muscle function in *mdx* mice was not adversely affected by the vibration training. Lastly, mice that were vibration trained had smaller subcutaneous fat pads and greater intramuscular triglyceride concentrations compared to non-vibrated mice. Combined, these data suggest that vibration training for 15 minutes per day, 5 days per week, for 8 weeks at 45 Hz and 0.6 *g* in rapidly growing mice does not significantly impact musculoskeletal function, but does affect fat.

### Trabecular bone

We hypothesized that 8 weeks of low intensity vibration training would improve trabecular morphology. Vibration training, however, did not affect any measure of trabecular morphology or dynamic histomorphometry in the proximal tibial metaphysis of wildtype or dystrophic mice (Figure 4 and Table 3). The anticipation of alterations in trabecular bone morphology was based on several reports of beneficial adaptations to bone in the proximal tibia of mice following vibration training. Specifically, improvements in trabecular thickness [39], [67], trabecular number [47], bone volume fraction [39], [47], [67], dynamic rates of bone formation [48], [67], and decreased trabecular separation [47] have been reported in bones of mice in response to 3 to 6 weeks of vibration training that had used similar low intensity parameters. In addition to these beneficial adaptations in healthy mice, vibration has also been shown to preserve or improve trabecular bone in mice modeling disuse [17], [68] and in patients with childhood diseases [22], [25], thus making vibration training an attractive therapeutic modality for DMD. The lack of vibration-induced alterations in trabecular bone in our study is not alone. Previous studies utilizing mouse models associated with physical inactivity and muscle weakness [41], [42], [43], as well as an uncontrolled, pilot study assessing the tolerability of high intensity vibration in DMD patients [28], also failed to detect alterations in trabecular or cortical bone density or serum markers of bone formation and metabolism from vibration training.

### Cortical Bone

Lower tibial bone ultimate load and stiffness in *mdx* mice compared to wildtype mice (Figure 5) are consistent with previous reports [33], [34] and have previously been attributed to altered bone geometry [34]. We hypothesized that 8 weeks of vibration training would improve cortical bone geometry and mechanical properties at tibia mid-diaphysis. Cortical bone, however, was not altered by vibration as indicated by the lack of any differences in cortical bone geometry or mechanical properties between vibrated and non-vibrated groups (Table 3 and Figure 5). These data are corroborated by evidence from others indicating that low intensity vibration did not alter bone geometry at the mid-diaphysis of the tibia [44], [45] and femur [14] in mice. Improvements in periosteal bone formation rate and mineral apposition rate at the tibial mid-diaphysis following vibration have been noted [44]. However, this increase in bone growth did not translate to improvements in cortical bone area, ultimate load, or stiffness. Cortical bone dynamic histomorphometry was not measured in the present study due to the lack of observed improvements in cortical bone geometry and mechanical properties.

The lack of an anabolic response in cortical and trabecular bone with vibration training in the present study may be attributed to multiple factors including vibration protocol parameters, transmission of the vibration stimuli to the musculoskeletal tissues, or the use of relatively young mice. Bone’s response to vibration is not universally effective and has been shown to preferentially respond to certain vibration stimuli [39], [40], [44], [48]. Therefore, it is possible that the vibration parameters utilized in the present study are optimal for eliciting an osteogenic gene expression response after 14 days of training [46], but not optimal for altering tibial bone strength and structure with long-term training. Bone’s response to vibration is also dependent upon how well the vibration stimuli are transmitted to the tissues of interest. Skeletal regions closest to the source have more robust responses [40] compared to distal sites where transmission is diminished [56], thus longitudinal growth of the tibia may have altered the magnitude transmission over the 8-week course of the study. Transmission of the stimulus is also influenced by muscle activation patterns and joint angles [56], [69]. These factors were not controlled for in the present study, however mouse behavior and posture while on the platform did not appear to be altered over 8 weeks of training. It is possible that in mice, a higher intensity vibration (i.e., accelerations exceeding 1 *g*) might better amplify transmission and provoke an osteogenic response as previously shown [44]. Lastly, it is plausible that the use of young, growing mice in the present study masked our ability to quantify the efficacy of vibration to improve bone. Between 3–11 weeks of age, the rate of longitudinal bone growth is maximized in mice, and therefore may have a ceiling effect at which the bone becomes unable to respond to additional mechanical stimuli.

### Skeletal Muscle

Eight weeks of vibration training did not alter any measure of hindlimb muscle functional capacity or structure (Figures 2 and 3 and Tables 2) and therefore our results do not support the notion that low intensity vibration is of benefit to muscle. The overall efficacy of low intensity vibration to improve muscle function in humans remains controversial [23], [24], with various reports of beneficial effects [21], [22], [25], [26], [27] and those reporting lack of alterations [28], [29]. Few studies have used mouse models to investigate vibration and skeletal muscle and those reports are also inconsistent in regard to effects on muscle size [30], [41], [49], [50]. The vibration training protocol used in the present study did not improve muscle size or strength in *mdx* or wildtype mice. The lack of vibration-induced improvements in muscle is consistent with results from another study that used botulism toxin to induce muscle weakness [41], but contradicts our previous vibration work in wildtype mice in which muscle strength improved by 10% despite no effect on muscle mass, size, or protein content [50]. Of interest, our previous study on wildtype mice was conducted using the same vibration device except that the vibration parameters were slightly different (1.0 *g* and 45 Hz) and the device was placed on a bench top [50]. In subsequent studies [46], [70] and the current study, our device was mounted on a concrete vibration-isolation base, which reduced the error between actual and target acceleration to ±0.37% [70]. This modification was intended to minimize the variation in acceleration produced by the vibration device. It is possible that the homogenous acceleration stimulus in the present study may be responsible for preventing the improvements in muscle strength we previously observed.

Contraindications of vibration on muscle have been reported [30], [31], and due to the high susceptibility of dystrophic muscle to injury, it was necessary to establish that vibration is a safe training modality. Our results show that 8 weeks of low intensity vibration training was not deleterious to any measure of muscle functional capacity (Figures 2–3 and Tables 2). The lack of injury with vibration training corroborates our previous findings in healthy mice [50] and preliminary data in patients [28], and contradicts the two studies which have reported muscle-specific contraindication of vibration (i.e., reduced vascularity in the distal soleus muscle in response to a low intensity vibration [30], and centrally-located nuclei in muscle fibers following relatively high intensity vibration (i.e., accelerations exceeding 1 *g*) [31]. Our thorough investigation utilized established recommendations for pre-clinical testing in *mdx* mice including a combination of *in* *vivo* and *ex* *vivo* assessment of muscle functional capacity providing a comprehensive evaluation of a training modality’s efficacy and safety [71]. We further complemented these data with histological analyses and plasma creatine kinase activity to confirm that vibration was not injurious to dystrophic muscle. Our results show that low intensity vibration training does not adversely affect dystrophic mouse muscle.

### Fat Pads and Intramuscular Triglyceride Concentration

Vibrated mice had smaller subcutaneous fat pad masses following 8 weeks of training (Figure 1). This vibration-induced reduction in fat mass has been consistently reported in rodents [47], [50], [54] and vibration training has even been shown to inhibit diet-induced obesity in mice [47]. To determine if vibration training also reduced intramuscular fat, we chose to measure triglyceride concentrations within the gastrocnemius muscle as this is a direct measure of muscle adiposity. The same approach has been utilized to measure triglyceride concentrations in mouse serum, liver and epididymal fat pads following 6 weeks of vibration [47], however we are the first to investigate intramuscular triglycerides. Specifically, we showed that vibration-trained mice had intramuscular triglyceride concentrations that were 26% higher than control mice (Table 1). This finding contrasts the earlier report that triglyceride concentrations were not different in the blood, liver or fat pads [47]. The physiological relevance of the vibration-induced increase in intramuscular triglycerides is not clear. Elevated intramuscular triglyceride concentration has been associated with metabolic disease, however, it also increases in response to exercise training [72]. This latter non-pathological response could potentially be an advantageous adaptation induced by vibration training, but more work will need to be done. Our previous work did show that 8-weeks of vibration-induced reductions in fat were not attributed to alterations in either energy balance (i.e., food intake and physical activity) [46], [50] or mitochondrial enzyme activity (i.e., of nicotinamide adenine dinucleotide-tetrazolium reductase reactivity) [50]. An alternative mechanism suggests that vibration may influence bone marrow cells’ lineage commitment away from adipocytes toward the osteoblast lineage [16], [17], [47]. This was based on the finding that vibrated mice had increased expression of the adipogenic gene, PPARγ (27%) and reduced expression of the transcription factor Runx2 (73%) [47]. Combined, our results indicate that vibration training influences fat distribution in mice.

In conclusion, the present study has established that 8 weeks of low intensity, high frequency vibration training for 15 min per day, 5 days per week at 45 Hz and 0.6 *g* did not significantly impact trabecular or cortical bone within the tibia of young, growing *mdx* or wildtype mice. Hindlimb muscle functional capacity was also not affected, implying that this type of vibration is safe for dystrophic muscle and would likely not have deleterious effects on disease progression. Vibration training may aid in slowing the acquisition of fat mass and how this could impact the progression of this or other diseases is interesting to consider. Collectively, our results do not support the idea that vibration training could be an effective modality for improving bone or muscle in the context of a muscle disease, but further research is needed to determine if alternative combinations of vibration parameters or a prolonged duration of training, or perhaps using an adult mouse model, could elicit beneficial musculoskeletal functional responses.

## Supporting Information

Video S1 **Vibration stimulus was well tolerated by 3-week old** ***mdx*** **and wildtype mice.** Behaviors, ambulation patterns and activities were indistinguishable between 3-week old wildtype mice (n = 2 mice on the left) and *mdx* mice (n = 2 mice on the right).
(WMV)

Video S2 **Vibration stimulus was well tolerated by 11-week old** ***mdx*** **and wildtype mice.** Behaviors, ambulation patterns and activities were indistinguishable between 11-week old wildtype mice (n = 2 mice on the left) and *mdx* mice (n = 2 mice on the right).
(MP4)
